# Maternal DHA Supplementation and Fetal Brain Development: Benefits, Risks, and Individual Needs

**DOI:** 10.3390/nu18203438

**Published:** 2026-10-20

**Authors:** Emma Whatley, Abigail Frommack, Emily Bayliss, Merritt Smith, Seth Woodfin, Ersilia Mirabelli, Dane Fausnacht, Kayla R. Gilley-Connor, William Moore

**Affiliations:** 1 Department of Biology and Chemistry, School of Health Sciences Liberty University, Lynchburg, VA 24515, USA; ewhatley1@liberty.edu (E.W.); angatz@liberty.edu (A.F.); mlsmith60@liberty.edu (M.S.); emirabelli@liberty.edu (E.M.); kgilley@liberty.edu (K.R.G.-C.); 2 Department of Biochemistry and Molecular Biology, F. Edward Hébert School of Medicine, Uniformed Services University of the Health Sciences, Bethesda, MD 20814, USA; emily.bayliss@usuhs.edu; 3 Department of Biomedical Sciences, West Virginia School of Osteopathic Medicine, Lewisburg, WV 24901, USA; swoodfin@osteo.wvsom.edu; 4 Department of Biology, School of Sciences and Agriculture, Ferrum College, Ferrum, VA 24088, USA; dfausnacht@ferrum.edu

**Keywords:** omega-3 fatty acids, DHA, fetal neural development, maternal diet, nutrition

## Abstract

Maternal nutrition is a key determinant of fetal development, particularly in the context of neural regulation and maturation. Omega-3 polyunsaturated fatty acids, especially docosahexaenoic acid (DHA), play an essential role in neural differentiation, synaptogenesis, myelination, and neuroprotection. While the neurodevelopmental benefits of DHA are well-documented, the effects of excessive maternal DHA intake and the potential long-term consequences on offspring neurophysiology remain poorly understood. The purpose of this review is to synthesize current findings on the role of DHA in fetal neural development, emphasizing biochemical mechanisms and developmental outcomes. It further explores emerging concerns associated with excessive intake, the varied responses to intake, the varied sources of supplements, and the influence of maternal metabolic conditions, such as diabetes, on DHA bioavailability and fetal brain development. Finally, the review highlights the need for individualized supplementation strategies in maternal nutrition and considers the potential epigenetic implications of prenatal DHA exposure, to ultimately inform future research and therapeutic strategies in maternal–fetal health and neurodevelopmental biology.

## 1. Introduction

**Figure 1:**
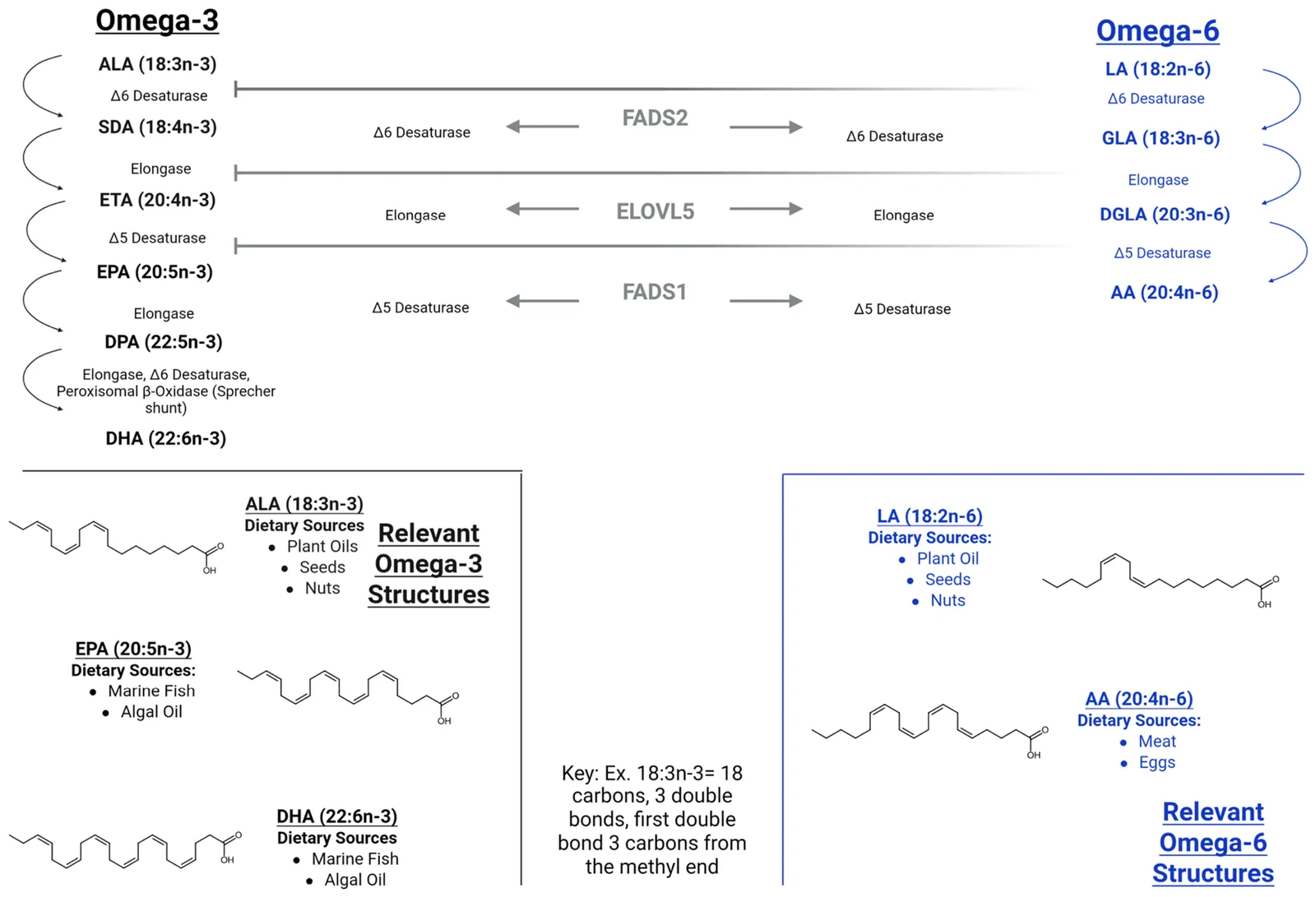
Biosynthetic relationships and structures of the principal omega-3 and omega-6 polyunsaturated fatty acids discussed in the review. α-Linolenic acid (ALA, 18:3*n*-3) and linoleic acid (LA, 18:2*n*-6) are converted through parallel, enzymatically analogous pathways to eicosapentaenoic acid (EPA, 20:5*n*-3) and docosahexaenoic acid (DHA, 22:6*n*-3), or to arachidonic acid (AA, 20:4*n*-6), respectively, via shared Δ6-desaturase, elongase, and Δ5-desaturase activity (FADS2, ELOVL2/5, FADS1). Curved arrows indicate sequential enzymatic conversion steps within each pathway; horizontal arrows and connecting lines indicate that the same enzyme (FADS2, ELOVL2/5, or FADS1) catalyzes the corresponding step in both the omega-3 and omega-6 pathways. Because the omega-3 and omega-6 pathways compete for the same desaturase/elongase pool, high dietary intake of LA can suppress ALA-to-DHA conversion. Dietary sources are indicated for each fatty acid; EPA and DHA are also obtainable preformed from marine or algal sources, bypassing the need for conversion from ALA. Structures were drawn from SMILES using RDKit (version 2026.03.6) with identical drawing parameters (bond length, line width, font, orientation, and carboxylic acid representation) and verified against the Merck Index [8]. All double bonds are cis (Z). The nomenclature key explains the *n*-3/*n*-6 shorthand used throughout the manuscript. Created in BioRender. Smith, M. (2026) https://BioRender.com/7ss5tc8 (accessed on 16 October 2026).

Omega-3 fatty acids are polyunsaturated fatty acids (PUFAs) that are essential for numerous biological processes, particularly those related to brain health [1,2,3,4]. Many omega-3 fatty acids cannot be synthesized endogenously, so adequate dietary intake is critical. The three primary omega-3 fatty acids are α-linolenic acid (ALA), eicosapentaenoic acid (EPA), and docosahexaenoic acid (DHA). ALA is found in plant-based sources such as nuts, seeds, and vegetable oils, and can be endogenously converted to EPA and DHA, though the process is notably inefficient [5,6] (Figure 1). By contrast, EPA and DHA are predominantly obtained from marine sources, with DHA constituting over 40% of neural PUFAs [7].

**Figure 2:**
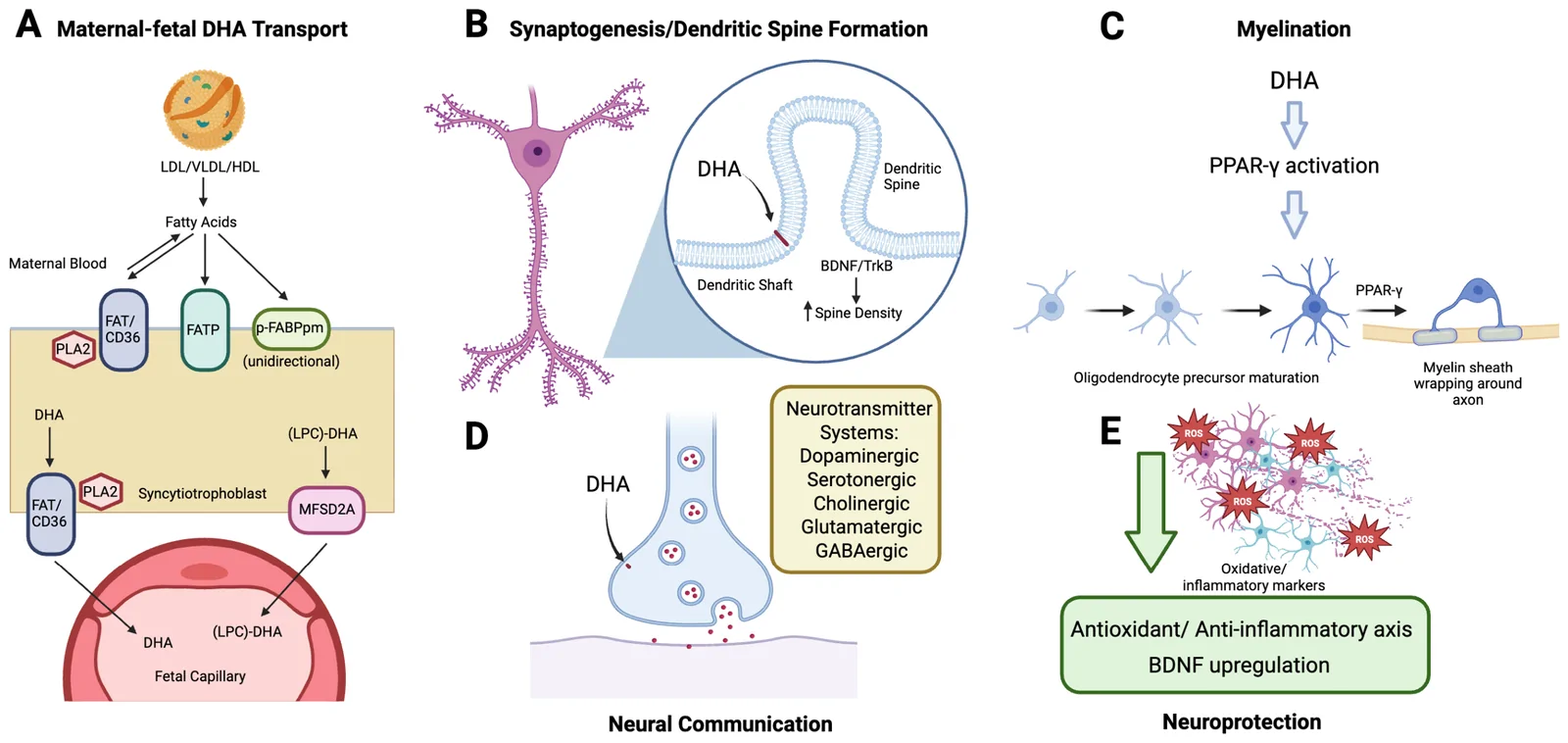
Mechanistic basis for the effects of DHA on fetal brain development. (**A**) Maternal-to-fetal DHA transport across the placenta. DHA is delivered to the maternal side of the syncytiotrophoblast bound to VLDL/LDL/HDL, released as free fatty acid by FAT/CD36 and phospholipase-A2 (PLA2), and transferred into the syncytiotrophoblast via FATP and P-FABPpm, with P-FABPpm conferring the unidirectional, maternal-to-fetal directionality of this transfer. Within the syncytiotrophoblast, DHA is re-exported basally as free DHA (via FAT/CD36 and PLA2) and as LPC-DHA (via MFSD2A) into the fetal capillary. (**B**) Synaptogenesis and dendritic spine formation. DHA incorporates into the neuronal membrane phospholipid bilayer, a structural role that supports dendritic spine budding from the dendrite shaft; BDNF/TrkB signaling drives the resulting increase in spine density. (**C**) Myelination. DHA promotes oligodendrocyte precursor maturation via PPAR-γ activation, driving myelin sheath formation around the axon. (**D**) Neural communication. DHA embedded in the presynaptic membrane modulates multiple neurotransmitter systems, including dopaminergic, serotonergic, cholinergic, glutamatergic, and GABAergic signaling systems. (**E**) Neuroprotection. DHA supports neuronal survival by blocking oxidative and inflammatory damage through a combined antioxidant/anti-inflammatory axis and BDNF upregulation. Arrows indicate the direction of transport (**A**), incorporation of DHA into membranes (**B**,**D**), or the progression of steps (**C**,**E**); the upward arrow in (**B**) indicates increased spine density. Created in BioRender. Bayliss, E. (2026) https://BioRender.com/uqq97py (accessed on 16 October 2026).

DHA plays a crucial role in neural differentiation, neuritogenesis, synaptogenesis, neuroplasticity, and signal transduction (Figure 2) [7,9]. DHA supports neurodevelopment throughout childhood and contributes to cognitive function maintenance throughout life [10,11,12], but its influence is particularly pronounced during fetal development. During this critical period, DHA supports central nervous system (CNS) development by exerting antioxidant and anti-inflammatory effects systemically and by contributing to the structural integrity of neuronal phospholipid membranes [13]. Importantly, because the fetus is largely dependent on maternal DHA, which is actively transported across the placenta via efficient transporters such as fatty acid transport proteins (FATPs), especially major facilitator superfamily domain-containing protein 2A (MFSD2A), mutations affecting those transporters can lead to pathological outcomes in the developing fetus [14,15].

Omega-3 fatty acid supplementation is widely recognized as beneficial. In adults with low blood DHA and in aging individuals, supplementation has been shown to improve cognition [16]. It is also well understood to be beneficial for both maternal and fetal health; however, its specific roles in modulating genetic, metabolic, and epigenetic pathways remain unclear [13,17]. This review aims to summarize existing knowledge, discuss ongoing controversies, and highlight critical gaps in the literature to guide future research in maternal–fetal nutrition and neurodevelopmental biology. While our central focus is prenatal maternal DHA supplementation and its consequences for fetal brain development, we also draw on postnatal maternal supplementation during lactation, breast milk and infant formula composition, and animal and mechanistic studies where these clarify biological pathways or extend the developmental window under discussion; these different forms of exposure are distinguished explicitly throughout, including in Table 1.

Several prior reviews have addressed aspects of maternal DHA intake and offspring neurodevelopment, each of which has a narrower or differently focused scope than the present work. The clinical implications of DHA supplementation have been broadly summarized across pregnancy and childhood [13]. Domain-specific RCT evidence (visual/cognitive function and language development) has been synthesized [28,29]. Perinatal PUFA intake in relation to neurodevelopmental disorders has been generally reviewed [30], as has the association between maternal DHA status and infant neurodevelopmental outcomes [31]. More recently, a 2025 review examined omega-3 fatty acids in fetal brain development with an emphasis on cognitive mechanisms and pediatric depression outcomes [32]. While these works establish the general benefits of DHA and outline the RCT evidence base, none integrates the biochemical transport and signaling mechanisms with the emerging evidence for dose-dependent adverse effects, individual genetic and metabolic variability (i.e., FADS2 polymorphisms, maternal diabetes), and the epigenetic implications of prenatal DHA exposure within a single synthesis. The present review addresses this gap. To our knowledge, it is the first to integrate these strands into a single synthesis that explicitly weighs benefits against dose-dependent risks, stratifies the evidence base by exposure route (Table 1) to avoid conflating mechanistically distinct exposures, and argues from that risk evidence toward a specific individualized-dosing research agenda, rather than a uniform-benefit narrative.

### Literature Search Strategy

Literature for this narrative review was identified primarily through PubMed and Google Scholar, supplemented by direct access to journal websites when full text was not readily available through PubMed. Searches used combinations of terms relevant to each topic that is addressed in the review, including “docosahexaenoic acid,” “DHA,” “maternal supplementation,” “fetal brain development,” “placental fatty acid transport,” “MFSD2A,” “FADS2,” and “omega-3 fatty acids AND pregnancy outcomes,” with additional sources identified through screening the references lists of key articles. No date restriction was applied and sources ranged from foundational studies to those published in 2025. Articles were included if they addressed maternal or fetal omega-3/DHA metabolism, placental transport, neurodevelopmental outcomes, or related mechanisms in human or relevant animal models, and were excluded if not peer-reviewed or not published in English. This review is narrative rather than systematic and does not follow a predefined protocol or PRISMA methodology. Articles were selected based on relevance to the biochemical and clinical themes discussed. Human clinical and observational studies were prioritized as the primary evidence for neurodevelopmental outcomes; animal and in vitro mechanistic studies were included specifically to illustrate biological pathways not yet characterized in humans and are identified as preclinical evidence throughout (see Table 1’s Study Type column).

## 2. Biochemical Mechanisms of Omega-3 Fatty Acids in Fetal Neural Development

To get metabolically available DHA in the bloodstream, DHA must be synthesized from precursor ALA or directly consumed. Omega-3 fatty acids are primarily consumed as ALA and converted to EPA and DHA. However, the conversion rate is highly inefficient (<1%) due to metabolic competition with omega-6 fatty acids [33,34,35] (Figure 1). Maternal DHA status/intake has been positively associated with specific infant neurodevelopmental outcomes, problem-solving at 12 months, and visual acuity toward the end of the first postnatal year [36,37]. Furthermore, the fetal capacity to synthesize DHA is limited due to developmentally limited lipid synthesis, underscoring the importance of maternal dietary intake. Maternal DHA is preferentially taken up by placental lipoprotein lipase. Through stable isotope techniques, it has been suggested that very-low-density lipoproteins (VLDL), low-density lipoproteins (LDL), and high-density lipoproteins (HDL) provide the fatty acids to the placenta, and which are then transferred via phospholipase-A2 and intracellular lipases and fatty acid translocase, FAT/CD36 [30,38]. DHA is transported then across the cell membrane via FATPs and lipoproteins. These transporters can work in either direction, but the placental fatty acid-binding protein (P-FABPpm) favors the unidirectional flow of fatty acids from mother to fetus. Free DHA and lysophosphatidylcholine (LPC)-DHA act as fetal sources for MFSD2A [30]. Notably, MFSD2A serves as the primary transporter for DHA to cross the blood–brain barrier (Figure 2A), owing to its high affinity for DHA. This has been confirmed by studies showing that reduced MFSD2A expression in APOE4-TR and E4FAD transgenic mice results in severe DHA deficiency [7,39]. In clinical practice, impaired placental lipid transport, whether from maternal dyslipidemia, diabetes, or genetic variation in MFSD2A, can manifest as intrauterine growth restriction or subtle neurocognitive delay, reinforcing the translational importance of efficient DHA transfer mechanisms [14,40]. Furthermore, deletion of MFSD2A leads to microcephaly and hypomyelination in humans, further emphasizing the importance of DHA [30]. Consistent with these findings, maternal DHA status and placental function have been shown to be critical for fetal DHA supply, given the limited capacity of the fetus and placenta to synthesize long-chain polyunsaturated fatty acids [41].

Because maternal DHA intake is a key determinant of fetal DHA availability, physiological changes during pregnancy, dietary consumption, and maternal genetic factors influence its uptake and distribution [42]. During pregnancy and lactation, lipid metabolism is greatly enhanced to meet both maternal and fetal demand, which increases the need for PUFA intake [7]. The *FADS1* and *FADS2* (fatty acid desaturase) genes encode rate-limiting enzymes involved in PUFA biosynthesis, and polymorphisms in these genes have been associated with metabolic conditions, including diabetes. Elevated maternal DHA intake, especially in cases of *FADS2* polymorphism, may mitigate fetal DHA deficiency by compensating for reduced enzymatic efficiency [43].

DHA accumulation in the fetal brain peaks during periods of rapid synaptogenesis, a central process to neural development, and gray matter expansion [44]. It plays a pivotal role in neuronal signaling, dendritic spine formation, and synaptic plasticity [31]. DHA enhances acetylcholine release, which is critical for long-term neuroplasticity [45,46], and its deficiency has been linked to mood disorders such as depression [47]. In addition, DHA promotes oligodendrocyte maturation, and in turn directly stimulates the formation of myelin sheaths in the CNS [48] (Figure 2C). The myelin is composed of proteins and lipids that insulates the axons to facilitates rapid and efficient electrical signal transmission along the nerve cell. Aberrant myelination or loss of myelin is implicated in neurological disorders, including multiple sclerosis [49]. DHA supplementation has been shown to increase myelination of axonal bundles in offspring [50]. Moreover, elevated levels of DHA in the maternal diet have been associated with increased dendritic spine density in the hippocampus of offspring (Figure 2B), which is a marker of synaptic connection and, in turn, enhanced cognitive and emotional development [51,52]. Abnormalities in the dendritic spine density in the hippocampus can be linked to the cognitive impairments in schizophrenia and autism spectrum disorder (ASD) [53,54]. Prenatal DHA supplementation has been shown to positively affect infant attention/regulation of state across the first year, and higher-dose prenatal DHA is associated with more rapid visual learning/attention on habituation tasks [55,56].

DHA also regulates the expression of brain-derived neurotrophic factor (BDNF), an essential neurotrophin responsible for the maintenance of neurons and their synaptic transmission and, in turn, higher functions such as learning and memory [57] (Figure 2E). Reduced levels of BDNF have been associated with systemic challenges including hearing impairment, coronary heart disease, and panic disorder [58,59,60]. From a clinical standpoint, DHA’s modulation of neurotrophins such as BDNF may underlie observed reductions in maternal postpartum depression and improved infant affect regulation, underscoring its dual relevance to obstetric and neuropsychiatric health. Omega-3 supplementation has shown significant improvements in depressive symptoms in perinatal women, and independently, prenatal DHA has been reported to improve infants’ attention and regulation of state in the first year [55,61]. Notably, individuals with Alzheimer’s disease often exhibit reduced BDNF expression [62]. In an animal model of traumatic brain injury (TBI), an increased intake of DHA was sufficient to reduce the oxidative damage and the learning disabilities recorded in rats after the injury [63,64]. Furthermore, higher prenatal DHA exposure is associated with increased BDNF levels, suggesting a neuroprotective role in fetal brain development due to the nature of BDNF increasing cell proliferation and differentiation [57,65,66]. Because DHA is mostly found within the synaptic membrane, it interacts with several neurotransmitter systems that are critical for mood regulation and cognitive performance, including the dopaminergic, serotonergic, cholinergic, glutamatergic, and gamma-aminobutyric acid (GABA)ergic neurotransmitter systems [67,68] (Figure 2D), with more limited evidence also pointing to modulation of noradrenergic and endocannabinoid signaling [68]. While glutamate and GABA are the primary excitatory and inhibitory neurotransmitters supporting cognitive and affective processes, both also play extensive roles outside these domains, including in sensory and motor function. The dysregulation of these systems is implicated in a variety of neurodevelopmental disorders [69,70]. For example, elevated levels of DHA have been shown to elicit antidepressant effects, potentially through enhanced serotonin (5-HT) transport by endothelial cells [71,72].

## 3. Maternal and Fetal Variability in DHA Availability During Pregnancy

Beyond diet, maternal health and lifestyle factors have a significant impact on DHA levels, which can influence fetal development. Substantial interindividual variability exists in maternal and fetal docosahexaenoic acid (DHA) status during pregnancy. Observational studies consistently demonstrate several-fold differences in maternal plasma, erythrocyte, and cord blood DHA concentrations among pregnant women, even within populations consuming broadly similar diets [73,74,75]. These differences arise from the combined influence of dietary intake, endogenous fatty acid metabolism, placental transport capacity, and maternal metabolic health. Stable isotope and lipidomic analyses indicate that circulating DHA availability during late gestation is particularly sensitive to maternal physiological state, reflecting dynamic regulation of lipid synthesis, mobilization, and trafficking [76,77]. Such variability is biologically meaningful, as fetal DHA accretion accelerates during the third trimester, coinciding with rapid brain growth and neural membrane expansion [31].

Dietary DHA and its precursor α-linolenic acid (ALA) undergo tightly regulated absorption and metabolic processing before becoming available to the fetus. Following intestinal absorption, ALA and DHA are incorporated into chylomicrons and delivered to the maternal liver, where they are either oxidized, stored, or repackaged into VLDL [78]. Hepatic elongation and desaturation of ALA to eicosapentaenoic acid (EPA), docosapentaenoic acid (DPA), and ultimately DHA require coordinated activity of Δ5- and Δ6-desaturases encoded by the FADS gene cluster [79]. Conversion efficiency is inherently low in humans and exhibits marked interindividual variability, making fetal DHA supply highly dependent on maternal intake of preformed DHA [79,80]. Placental transfer further refines fetal exposure through selective uptake mechanisms that favor long-chain polyunsaturated fatty acids, particularly DHA, over other circulating lipids [41].

Maternal stress and adverse metabolic states represent major non-dietary factors that reduce DHA availability to the fetus. Dysregulated lipid metabolism has even been identified as a marker of preterm birth [81]. Obesity, insulin resistance, and gestational diabetes mellitus are associated with chronic low-grade inflammation, elevated circulating cytokines, and impaired insulin signaling, all of which suppress hepatic desaturase activity and favor fatty acid oxidation over elongation and storage [82,83,84]. Increased dietary linoleic acid intake exacerbates this effect by directly competing with ALA for Δ6-desaturase, further reducing endogenous DHA synthesis and shifting lipid mediator profiles toward pro-inflammatory pathways [79,85] (Figure 1). Human studies consistently report lower plasma and erythrocyte DHA concentrations in pregnant women with obesity or metabolic dysfunction compared with metabolically healthy controls, independent of reported DHA intake [83,84].

Placental lipid handling is also directly altered by stress-related metabolic disturbances. The syncytiotrophoblast expresses multiple fatty acid transporters, including FAT/CD36, FATPs, and plasma membrane fatty acid-binding protein, which coordinate maternal–fetal DHA transfer [41,86]. Maternal obesity and metabolic stress impair placental lipid metabolism by reducing in beta-oxidation and altering the expression of FATPs [86]. A particularly important pathway involves LPC-bound DHA transported via the sodium-dependent transporter MFSD2A [87]. Reduced MFSD2A expression has been documented in placentas from pregnancies complicated by gestational diabetes, and this reduction is associated with lower cord blood DHA concentrations [88]. Oxidative stress and lipid accumulation within placental tissue further impair transporter trafficking and intracellular lipid remodeling, effectively limiting DHA export to the fetal circulation even when maternal DHA intake is adequate [84,88].

Genetic polymorphisms within the FADS gene cluster significantly influence baseline DHA synthesis capacity and modify responsiveness to both dietary ALA and DHA supplementation contributing to variability in fetal DHA exposure [79,89]. Hormonal changes during pregnancy, including fluctuations in estrogen, cortisol, and thyroid hormones, regulate hepatic lipid metabolism and placental transporter expression, providing endocrine pathways through which psychosocial stress may indirectly affect DHA delivery [77,90]. Activation of the maternal hypothalamic–pituitary–adrenal axis increases glucocorticoid signaling, which promotes fatty acid oxidation and impairs insulin sensitivity, thereby reducing the circulating DHA pool available for placental transfer [90]. Environmental stressors such as cigarette smoke exposure have also been associated with lower maternal and cord blood DHA concentrations, likely through oxidative and inflammatory mechanisms [91,92].

Maternal DHA levels are even more critical considering the fetus has a limited capacity to synthesize DHA from ALA. Stable isotope studies in preterm and term infants demonstrate minimal conversion of ALA to DHA, with most ALA undergoing oxidation or conversion to intermediate metabolites such as EPA or DPA [80,93]. Placental and fetal tissues express relatively low levels of elongase and desaturase enzymes compared with maternal liver, further constraining endogenous DHA production [78]. Consequently, fetal DHA accretion depends primarily on maternal DHA status and the efficiency of placental transport mechanisms rather than fetal synthesis [94].

Interventions that directly increase maternal DHA intake remain the most effective strategy for enhancing fetal DHA exposure. Randomized controlled trials consistently show that DHA supplementation in the range of 400 to 1000 mg per day increases maternal plasma and erythrocyte DHA concentrations and significantly elevates cord blood DHA at birth [56,74,95,96]. These effects are particularly pronounced in late gestation, when fetal DHA demand is highest. Complementary lifestyle interventions that improve insulin sensitivity, reduce systemic inflammation, and moderate excessive linoleic acid intake may further enhance DHA trafficking by restoring hepatic desaturase activity and placental transport capacity [79,83,85]. Together, these findings indicate that fetal DHA delivery reflects an integrated system involving maternal diet, metabolic health, endocrine regulation, and placental function rather than dietary intake alone.

## 4. Controversies and Differing Schools of Thought

The role of DHA in fetal neurodevelopment continues to be debated, due to a range of conflicting findings reported across studies (Table 1) [28]. While numerous investigations, spanning prenatal maternal supplementation, postnatal (lactation) supplementation, and infant formula trials, have demonstrated clear benefits of DHA exposure, particularly for early cognitive function and neuroprotection [18,22,24,27,97], other studies across these same exposure types report minimal or no measurable effects [19,20,21,23,25,29]. For example, long-term follow-ups with children whose mothers or themselves received DHA supplementation have sometimes revealed no significant differences in cognitive performance compared to non-supplemented controls, suggesting that genetic or environmental factors may confound outcomes [19,20,23,25].

There is also evidence that DHA intake during pregnancy is linked to a number of problems including gestational diabetes, intrauterine growth restriction, postpartum depression, preeclampsia, and preterm birth [98]. Findings on child outcomes have also been mixed, with one lactation-supplementation trial [19] and one infant-formula trial [26] each reporting a reduction in word comprehension in one-year-old children who had received DHA supplementation, and a seven-year follow-up of a randomized controlled trial finding that children of mothers who received prenatal DHA supplementation showed greater parent-reported behavioral problems and executive dysfunction than children in the placebo group [99]. Several methodological limitations may contribute to such variations. Many studies lack standardized baseline conditions, and participant populations often differ widely in genetic background, maternal nutritional status, socio-economic status, and socio-environmental exposure. In some cases, DHA supplementation may have corrected underlying deficiencies, such as low maternal DHA levels or FADS2 gene polymorphisms, thus bringing developmental outcomes from below-average to normative rather than enhancing performance above baseline. Future studies using twin cohorts could provide more controlled insight into the isolated effects of maternal DHA.

In the context of neurodevelopmental disorders such as attention-deficit/hyperactivity disorder (ADHD) and ASD, increased maternal omega-3 fatty acid intake has been associated with offspring symptomatic improvement in some cases [56,100]. However, evidence remains inconsistent. Some studies report no significant protection or change in ASD prevalence or severity, while the differences in study design, supplementation, duration, and dosage further confound the interpretation of outcomes [30,101]. Preclinical work offers some support for a protective role. In *Fmr1* knockout-mice, a model of Fragile X syndrome (the leading known monogenic cause of ASD), prenatal exposure to an omega-3 fatty acid-enriched diet improved isolation-induced ultrasonic vocalizations (USVs—one of the few measurable behavioral readouts in neonatal pups) in *Fmr1* heterozygous and homozygous females, though this effect was not observed in males [102]. Similarly, although increased DHA intake has been linked to reductions in depressive behavior and brain mass loss in an animal model of neonatal hypoxic–ischemic (HI) encephalopathy, the underlying mechanisms remain speculative and are subject to debate [103].

Notably, there is growing recognition of a potential dose-dependent effect of DHA, with both insufficient and excessive levels linked to adverse outcomes. DHA deficiency and impaired MFSD2A-mediated placental transport have been linked to microcephaly and hypomyelination, while separately, emerging evidence suggests that high-dose supplementation may also carry risk. For example, observational studies have reported associations between maternal serum DHA concentrations and offspring schizophrenia-spectrum outcomes [104], and separate animal studies suggest excess DHA exposure can produce aberrant cortical maturation [105]. However, neither directly demonstrates that high-dose human supplementation causes these outcomes. This is consistent with clinical data: a study reporting adverse fetal effects administered daily doses of 800 mg [99], nearly three times the widely cited minimum recommended intake of at least 200 mg/day for pregnant women [106]. Because this range reflects a target intake rather than an established upper safety limit, this comparison illustrates the gap between guideline-level intake and doses used in trials that report adverse effects, rather than establishing 800 mg/day itself as unsafe. Dose individualization is likely especially important for mothers with gestational diabetes, obesity, or lipid-transport abnormalities, as excessive intake may alter platelet aggregation or glycemic control [107,108]. This raises the possibility that at least some of the inconsistent or adverse findings reported elsewhere in the literature reflect confounded high-dose exposure rather than a true harmful effect of DHA itself. For instance, mothers with high dietary DHA intake, often via oily fish consumption, may also have elevated exposure to co-occurring contaminants such as per- and polyfluoroalkyl substances (PFAS), raising the question of whether some reported “high-dose” adverse effects are attributable to DHA or to unmeasured co-exposures. This remains speculative and would require work that directly assesses co-exposure.

Safety concerns also extend to the immunological and metabolic effects of excessive DHA intake. High doses of DHA may alter fetal immune responses or interfere with fatty acid metabolic pathways. Specifically, DHA directly inhibits desaturase activity at both the transcriptional and enzymatic levels. While this can help preserve the levels of DHA and EPA, excessive desaturase inhibition has been implicated in diseases such as non-alcoholic fatty liver disease, Alzheimer’s disease, schizophrenia, depression, cancer, and type 2 diabetes [109].

Finally, the source of DHA supplements is a growing area of concern. Omega-3 fatty acids are frequently derived from fish, which may contain environmental toxins, such as mercury, dioxins, furans, and polychlorinated biphenyls. These contaminants have been associated with neurological toxicity, immune suppression, and reproductive issues in women, thus raising questions about the safety of long-term supplementation using certain marine-derived products [110,111].

## 5. Current Research Gaps

Despite the general consensus that omega-3 supplementation is beneficial during pregnancy, emerging evidence suggests this view may be overly simplistic. In select cases, maternal DHA supplementation has been shown to produce adverse effects in the fetus, highlighting the need to consider individual genetic and metabolic differences. These findings suggest that supplementation may not be universally appropriate and that maternal genotype, particularly variations in fatty acid metabolism, could play a critical role in determining its benefit or harm. Further research is needed to better understand the implications of DHA intake in relation to neural processes that are implicated, and to clarify the risks associated with both deficient and excessive dosing. Because no upper safety threshold or optimal dose range has been established for maternal DHA intake, and because individual response likely varies with genotype and metabolic status, it may be worth investigating whether closer monitoring of maternal blood DHA levels during supplementation could help tailor dosing more safely, rather than relying on standardized intake recommendations alone. Additionally, the relationship between DHA and FADS gene polymorphisms remains poorly understood and warrants further investigation. Longitudinal studies tracking neurocognitive and behavioral outcomes into adolescence and adulthood are also needed to evaluate the sustained benefits or risks of prenatal DHA exposure.

Another significant gap in the literature concerns the epigenetic effects of omega-3 fatty acids. DHA has been shown to influence DNA methylation, potentially inducing hypermethylation in up to 52% of affected genes [112], though the biological consequences of these changes remain unclear. Similarly, ALA has been linked to hepatic epigenetic alterations [110,113]. The underlying mechanisms, specificity, and long-term impact of these modifications on offspring cognitive and metabolic outcomes have not yet been adequately explored. As such, the epigenetic landscape shaped by maternal omega-3 intake requires closer examination.

### Potential Future Developments

Future research should aim to identify individualized, optimal dose ranges for omega-3 fatty acids, accounting for maternal genotype, metabolic state, and pregnancy-associated conditions. One promising direction of interest includes investigating the long-term effects of prenatal DHA supplementation in the context of impaired lipid transport, which is often observed in maternal diabetes and related conditions [114]. Further, the broader biological effects of elevated maternal DHA, such as its influence on DNA methylation and histone modification, should be more precisely defined to assess their developmental relevance.

Given the concerns over environmental contaminants in fish-derived supplements, algae-derived DHA presents a promising alternative. Algal DHA has demonstrated superior bioavailability in infants [115], and is considered bioequivalent to DHA from salmon, which is a common source for fish oils [116]. The algae-derived supplements are also generally lower in environmental contaminants (i.e., mercury, PCBs) associated with some marine-derived products [117,118], making algal DHA a lower-contaminant-risk alternative worth further investigation, though direct comparative safety data in pregnancy remain limited. Industrial algae cultivation also allows for rigorous quality control and regulation and offers a potentially lower-contaminant option for maternal supplementation [119].

Maternal health is another important factor influencing fetal development. Omega-3 fatty acid supplementation has been shown to support gut microbiota, thereby mitigating stress-induced dysbiosis and promoting microbial stability [120]. This may, in turn, also influence fetal neurodevelopment through maternal immune modulation and reductions in chronic inflammation [121]. However, the specific neurodevelopmental consequences on the offspring remain underexplored.

An underappreciated but potentially important variable is the sex of the offspring. Some evidence from preclinical animal studies indicates sex-dependent responses to maternal omega-3 supplementation [102,122]. However, the extent and nature of those differences in humans are still unclear. Preliminary animal studies suggest male offspring may exhibit more pronounced responses than females [123,124], which raises the possibility of sex-specific dose recommendations. Further research should aim to elucidate how sex influences developmental interventions during pregnancy.

## 6. Conclusions

Maternal omega-3 fatty acid intake plays a critical role in supporting fetal neural development, with DHA acting as a key regulator of both structural and functional brain maturation. However, the optimal dosage, form, and long-term safety of supplementation remain incompletely defined. Preliminary evidence discussed in Section 4 raises the possibility that excessive intake, not just deficiency, could carry developmental risk, though this has not been established in humans and warrants targeted investigation. Similarly, individual variability driven by FADS gene polymorphisms, maternal metabolic status, and the sex of offspring suggests that a one-size-fits-all supplementation strategy may warrant reconsideration, a hypothesis that future dose-ranging and genotype-stratified studies should directly test.

Future research should examine whether integrating individual DHA assessment into prenatal nutrition counseling, combined with monitoring of maternal dietary patterns and fetal growth, could improve the safety and precision of maternal DHA supplementation. Future studies should also prioritize delineating optimal, genotype- and metabolically informed dosing strategies to allow clinical guidelines to more precisely balance the safety and efficacy of maternal DHA supplementation.

## Figures and Tables

**Table 1:** Summary of clinical studies examining the role of DHA in fetal and infant neurodevelopment organized by exposure type (prenatal maternal supplementation, postnatal/lactation supplementation, breast milk composition, or infant formula). Studies reporting on the same cohort across multiple publications (i.e., the Danish maternal fish-oil cohort [18,19]) are presented as a single row with outcome measures listed by timepoint rather than as independent entries. All studies listed measured DHA status in human subjects via erythrocyte or plasma composition; Study Type indicates whether each entry reflects a randomized controlled trial or observational design. Dose is reported as daily DHA intake (mg/day) where the original study specified it in these terms; NR (not reported) indicates that the dose was not available or reported only as a percentage of total fatty acids (%TFA), precluding direct mg/day comparison across studies.

Study	Study Type	Exposure Type	Population (*n*)	Duration	Technique	Effect	Dose
Helland et al. [20]	Clinical—RCT	Prenatal maternal supplementation	Children (143)	7 years	Cod liver oil vs. corn oil, from pregnancy week 18 through 3 months postpartum; KAB, IQ, BMI	No significant results in KAB, IQ, or BMI	1183 mg/day ^1^
Gibson et al. [21]	Clinical—RCT	Postnatal maternal supplementation (lactation)	Infants (52)	3 months	Breast milk; maternal supplement; DHA	IDC, but no long-term neurodevelopment effects	0–1300 mg/day ^2^
Hoffman et al. [22]	Clinical—RCT	Infant formula (DHA + AA-fortified)	Infants (61)	1 year	Breast milk, then DHA + AA-supplemented vs. commercial formula; RBC DHA; VEP	Supplemented infants had significantly more mature VEP acuity; no difference in stereoacuity	NR
Danish maternal FO cohort, Lauritzen et al. [18,19]	Clinical—RCT	Postnatal maternal supplementation (lactation)	Infants (97)	4 months	Breast milk; high FO intake; DHA; VEP	Infants with raised LCPUFA in blood showed higher VEP, but FO did not directly increase VEP	900 mg/day ^3^
			Infants (100) ^4^	9 months–2 years	FO maternal supplement; breast milk	No change in problem solving, passive vocabulary of FO lower at 1 year, but leveled out at 2	
Jensen et al. [23]	Clinical—RCT	Postnatal maternal supplementation (lactation)	Infants (230) ^5^	30 months	DHA; 4 months; BPD	IDC, but no advantages other than increased BPD at 30 months	200 mg/day ^6^
Agostoni et al. [24]	Clinical—Observational	Breast milk composition (observational)	Infants (55)	4 months	Breast milk; formula; LCPUFA-enriched formula DQ; DHA	IDC correlated with improved DQ score	NR
Jørgensen et al. [25]	Clinical—RCT	Infant formula (DHA-fortified)	Infants (37)	4 months	Breast milk; STF; DHAGF; DHAF; VEP	DHAGF gave IDC, but no statistical significance was achieved for VEP	NR
Scott et al. [26]	Clinical—RCT	Infant formula (DHA-fortified)	Children (274)	14 months	Formula; FO; egg; MBD; MCDI; VPCS	No change in MBD, DHA had significantly lower scores for MCDI and VPCS	NR
Birch et al. [27]	Clinical—RCT	Infant formula (DHA-fortified)	Infants (103)	52 weeks	Breast milk; LCPUFA supplement; DHA; VEP	Significantly improved VEP and stereoacuity, also IDC	NR

^1^ Dose of 10 mL cod liver oil daily; also contained 803 mg EPA/day. ^2^ Five-arm dose-ranging trial (0, 0.2, 0.4, 0.9, or 1.3 g DHA/d via algal oil); maternal supplement. ^3^ Dose of 4.5 g/d fish oil providing 1.5 g *n*-3 LCPUFA/d (60% DHA); Danish maternal FO-supplementation cohort (same trial, [18,19]). ^4^ *n* = 100 (9-month problem-solving assessment); language outcomes assessed at 1 and 2 years drew on smaller completer samples (*n* = 89 and *n* = 71 respectively) due to further attrition. ^5^ Total of 230 enrolled; 160 (83 DHA, 77 control) completed the 30-month follow-up for the primary outcome (Bayley PDI). ^6^ Maternal supplement, algal oil, via breast milk. Abbreviations: LCPUFA, long-chain polyunsaturated fatty acid; DQ, developmental quotient; DHA, docosahexaenoic acid; IDC, increased DHA concentration (erythrocyte/plasma); VEP, visual evoked potential; AA, arachidonic acid; RBC, red blood cell; FO, fish oil; STF, standard formula; DHAGF, formula containing 0.3 wt% docosahexaenoic acid and 0.5 wt% gamma-linolenic acid; DHAF, formula containing 0.3 wt% docosahexaenoic acid; BPD, Bayley Psychomotor Development Index; KAB, Kaufman Assessment Battery for Children; IQ, intelligence quotient; BMI, body mass index; MBD, Mental and Motor Scales of the Bayley Scales of Infant Development; MCDI, MacArthur Communicative Development Inventories; VPCS, Vocabulary Production and Comprehension Scales; NR, not reported.

## Data Availability

No new data were created or analyzed in this study. Data sharing is not applicable to this article.

## Abbreviations

The following abbreviations are used in this manuscript:

AA	Arachidonic acid
ADHD	Attention-deficit/hyperactivity disorder
ALA	α-Linolenic acid
ASD	Autism spectrum disorder
BDNF	Brain-derived neurotrophic factor
BMI	Body mass index
BPD	Bayley Psychomotor Development (Index)
CNS	Central nervous system
DGLA	Dihomo-γ-linolenic acid
DHA	Docosahexaenoic acid
DHAF	Formula containing 0.3 wt% docosahexaenoic acid
DHAGF	Formula containing 0.3 wt% docosahexaenoic acid and 0.5 wt% gamma-linolenic acid
DPA	Docosapentaenoic acid
DQ	Developmental quotient
EPA	Eicosapentaenoic acid
ETA	Eicosatetraenoic acid
FADS1/2	Fatty acid desaturase ½
FAT/CD36	Fatty acid translocase/cluster of differentiation 36
FATP(s)	Fatty acid transport protein
FO	Fish oil
GABA	Gamma-aminobutyric acid
GLA	γ-Linolenic acid
HDL	High-density lipoprotein
HI	Hypoxic–ischemic
IDC	Increased DHA concentration
IQ	Intelligence quotient
KAB	Kaufman Assessment Battery (for Children)
LCPUFA	Long-chain polyunsaturated fatty acid
LDL	Low-density lipoprotein
LPC	Lysophosphatidylcholine
MBD	Mental and Motor Scales of the Bayley Scales of Infant Development
MCDI	MacArthur Communicative Development Inventories
MFSD2A	Major facilitator superfamily domain-containing protein 2A
NR	Not reported
P-FABPpm	Placental plasma membrane fatty acid-binding protein
PFAS	Per- and polyfluoroalkyl substances
PUFA	Polyunsaturated fatty acid
RBC	Red blood cell
SDA	Stearidonic acid
STF	Standard formula
TBI	Traumatic brain injury
USV	Ultrasonic vocalization
VEP	Visual evoked potential
VLDL	Very-low-density lipoprotein
VPCS	Vocabulary Production and Comprehension Scale(s)
5-HT	Serotonin (5-hydroxytryptamine)
